# Behavioural entropy as an individual difference

**DOI:** 10.23889/ijpds.v8i3.2289

**Published:** 2023-09-11

**Authors:** Neo Poon, James Goulding, Anya Skatova

**Affiliations:** 1 University of Bristol; 2 University of Nottingham

## Introduction & Background

The availability of digital footprints data have provided new and invaluable opportunities for personality psychologists. One way to study individual differences with digital footprints data is through the lens of entropy, which is a measure of the degree of randomness of a probabilistic system. When applied to individual behaviour, entropy captures how predictable an individual’s (e.g., shopping) pattern of behaviour is over time. 

In this study, we proposed that entropy can be conceptualised as a proxy measure of Openness, a Big Five personality trait. We further studied entropy’s associations with external behavioural outcome, namely the voting outcomes of the 2016 EU ‘Brexit’ referendum in the UK. This referendum asked UK citizens whether the UK should stay in the EU (vote Remain) or leave the EU (vote Leave). It has been demonstrated that Leave (or ‘Brexit’) vote was heavily influenced by attitudes towards immigration which is associated with values of being less ‘open’ to other cultures, and therefore we expected that entropy – or tendency to try new things – would be associated positively with voting Remain.

## Objectives & Approach

With a massive data set (20,550,952 customers) provided by a large UK retail chain over a period of 2 years, we computed aggregated entropy for the Local Authority Districts (LADs). Further we investigated the relationships between entropy and personality traits, as well as between entropy and the referendum outcomes, at geographically aggregated levels.

## Relevance to Digital Footprints

This study brought together digital footprints data with external sources. This study also identified population level insights by examining personality traits and their utility in predicting sociopolitical outcomes.

## Results

Results of a linear regression model showed strong evidence supporting a positive relationship between entropy and Openness (b = 0.30, t = 3.30, p = .001), and a negative relationship between entropy and Neuroticism (b = -0.48, t = -3.53, p < .001).

Further, entropy was associated with outcomes of the EU referendum in each LAD. Results of another linear regression model showed strong evidence supporting a positive relationship between the percentage of Remain votes and entropy (b = 0.28, t = 4.80, p < .001).

## Conclusions & Implications

The relationship between Big Five trait Openness and entropy provided support that personality can be inferred from digital footprints data such as shopping history records. The positive relationship between entropy and the proportion of Remain vote demonstrated that people who are more open to new experiences voted Remain. Our findings have broader implications showing that it is possible to find associations between personality traits extrapolated from shopping data and real-world choices.

